# Muscle-Sparing Approach for Recurrent Hydatidosis of the Thigh and Psoas: Report of a Rare Case

**DOI:** 10.1371/journal.pntd.0000840

**Published:** 2011-01-25

**Authors:** Gaetano La Greca, Elia Pulvirenti, Salvatrice Gagliardo, Maria Sofia, Domenico Russello

**Affiliations:** Department of Surgical Sciences, Organ Transplantation and Advanced Technologies, University of Catania, Cannizzaro Hospital, Catania, Italy; New York University, United States of America

**Learning Points**

- Psoas and thigh, although rare localizations, could represent the site of primary or secondary hydatidosis.
- Muscular hydatidosis should always be considered in patients with previous history of such a disease, especially in cases of previous surgical dissemination.
- A muscle- or nerve-sparing attempt should always be performed in cases of wide diffusion as well.

## Presentation of Case

A 46-year-old male shepherd presented with a mildly painful mass 4.5 cm in diameter localized at the right groin and thigh, diffuse edema involving the right leg lasting for 2 weeks, and fever. The patient had had eight previous operations for diffuse hydatidosis with reported intraperitoneal seeding, but further information was unavailable.

**Figure 1:**
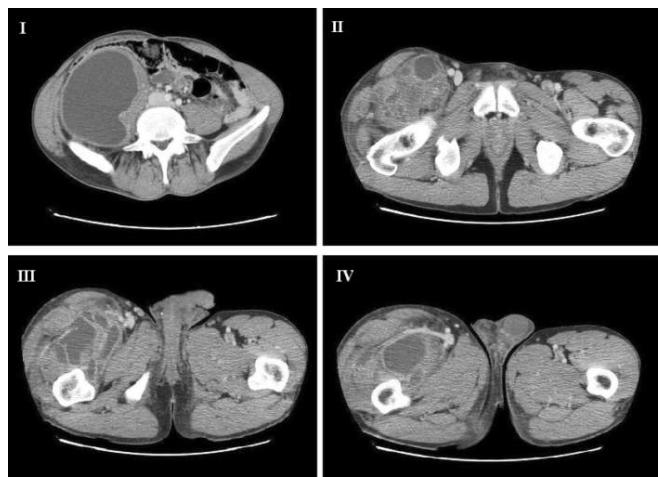
Preoperative basal computed tomography. The involvement of the psoas muscle and retroperitoneum from the Morrison's pouch up to the right iliac fossa and to the iliac region is evident (I, II). The same CT scan shows the involvement of the right thigh with the cysts localized deeply and behind the muscular aponeurotic plane of the femoral quadriceps and abductor muscles up to the knee (III, IV).

Laboratory tests are shown in Table 1. An abdominal ultrasound (US) and a Doppler US of the right leg detected the presence of multiple and partially confluent cysts localized up to the Scarpa's triangle. A computed tomography (CT) scan detected a multi-cystic 18-cm mass originating from the psoas muscle (Figure 1, I and II). Other cysts were localized deeply and behind the muscular aponeurotic plane of the femoral quadriceps and abductor muscles (Figure 1, III and IV). All these findings were suggestive of diffuse hydatidosis and the patient was promptly operated on with a muscle-sparing approach, for which a written consent was obtained.

**Table 1:** Patient's laboratory findings (normal values) at the admission.

White blood cells	12.5×10^9^/L (4–11.3×10^9^/L)
Neutrophil percentage	61.9% (41%–73%)
Eosinophil percentage	4.5% (1%–6%)
Red blood cells	5.12×10^12^/L (4.1–5.9×10^12^/L)
Hemoglobin	14.70 g/dL (13.5–18 g/dL)
Hematocrit	44.1% (40%–50%)
Alanine aminotransferase	20 U/L (14–63 U/L)
Aspartate aminotransferase	16 U/L (14–45 U/L)
Total bilirubin	0.9 mg/dL (<1.1 mg/dL)
Amylase	178 U/L (4–250 U/L)
Creatine kinase	190 U/L (40–175 U/L)
Lactate deydrogenase	467 U/L (240–450 U/L)
Erythrocyte sedimentation rate	12 mm/h (4–10 mm/h)

Piperacillin/tazobactam was administered from the date of admission to the day of surgery. Preoperative prophylaxis with benzimidazole derivatives was not performed due to the extent of the disease, the history of recurrences, and the need to perform the operation promptly to reduce the symptoms.

At surgery, the retroperitoneum was accessed and tissues surrounding the cysts were covered with sponges soaked with hypertonic saline. The cystic content was evacuated and the interior of the cyst was repeatedly washed with protoscolicides. Due to the tight adhesions with the peritoneal sac, only the lateral-lower portion of the cystic wall could be resected. No daughter cyst was found. Subsequently, the right thigh was anteriorly incised and the cysts were evacuated. The washing treatment was repeated but extensive resection was avoided to prevent any risk of unnecessary damage.

Histopathological examination did not detect viable protoscolices and routine cultures performed to individuate other pathogens were negative.

**Figure 2:**
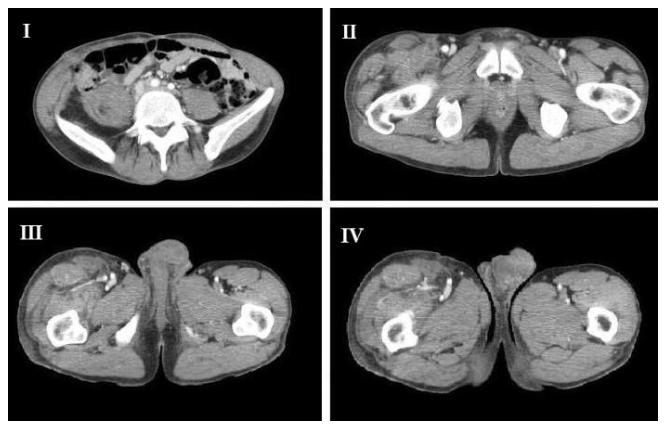
Postoperative computed tomography showing the outcome of the treatment. The psoas muscle has been spared despite the resection of the cysts (I, II); the right thigh is free of cysts with the conservation of the muscular structures (III, IV).

A CT scan performed before discharge showed the integrity of the psoas muscle (Figure 2, I and II) and the lack of residual cysts in the thigh with conservation of the muscular structures (Figure 2, III and IV). The patient was discharged after 11 days with 6 months of administration of mebendazole. Further investigations could not be performed because the patient missed the planned follow-up.

## Case Discussion

In secondary hydatidosis, whose reported incidence is between 1.1% and 25% [1], muscle involvement may represent a recurrence of previously treated disease [2]. The pathogenesis of this condition is different than primary hydatidosis, being related to the spilling of the cystic contents during a previous operation [1], together with the lack of post-operative administration of benzimidazoles [3], [4] and the lack of follow-up.

Hydatid cysts of the psoas and thigh are reported very rarely [2], [5]–[15]. Symptoms are generally related to cyst's dimension and location [16], and may include infection with fistulization in surrounding organs, and allergic reactions including anaphylactic shock.

Different types of serological tests may help in diagnosis [8]. In our case, no serological test was performed due to the strong clinical evidence related to patient's previous history and because of the need for prompt treatment.

US represents the preferred diagnostic method [9], [10]. CT is performed in cases of indeterminate US findings or negative immunological tests. Large dimensions, daughter cysts, and increased density of the hydatid membrane are pathognomonic [8]. Magnetic resonance imaging may clarify the diagnosis in some cases [11].

Medical therapy with a benzimidazole derivative is preferable whenever there is multi-organ involvement or when patients are not fit for surgery [9]. Although pre-surgical use of benzimidazoles may reduce the risk of recurrence and facilitate the operation, neither the required duration of such treatment, nor its efficacy, have been documented [17]. We decided not to administer pre-operative albendazole and to operate 1 week later, as suggested by another study [18]. Following surgery, we initiated treatment with post-operative administration of benzimidazoles [17].

Abnormalities in liver function, leucopenia and alopecia are side effects of the benzimidazoles [19]. Alopecia seems to be more frequently described after albendazole administration [20]. The onset of this complication induced our patient to discontinue the previous postoperative therapy and not to restart the administration of albendazole as suggested, so mebendazole was proposed as the only chance to prevent further recurrences.

The treatment of choice is complete surgical excision of the cyst and irrigation of the surrounding tissues with hypertonic saline. World Health Organization guidelines suggest several specific approaches for classic hydatidosis, but there is no mention regarding unusual localizations [17]. For cysts localized in the psoas muscle, the extraperitoneal approach represents the safest access to avoid peritoneal dissemination [9], [10]. The cysts should be removed without sacrificing any organ, but sometimes it is impossible because of the involvement of several organs or vital structures [7], [14]. Partial perycistectomy and cyst drainage seems to be the best approach due to its minimal invasiveness, effectiveness, and tolerability [15].

An alternative to surgery is the US-guided cyst puncture and injection of protoscolicides (puncture, aspiration, injection, and reaspiration [PAIR]), indicated for inoperable patients [17] and in cases of relapse after surgery [21]. However, in this specific case, PAIR could have been useful only as a first approach to reduce edema and inflammation.

Since our patient had eight prior operations with recurrence each time, perhaps none of these operations was adequately performed and the post-operative treatment was not properly instituted or monitored. The contemporary excision of all the macroscopic localization, the careful protection of the surgical field, and the postoperative administration of mebendazole distinguish our intervention from the eight that preceded it.

## The Presenting Case

This report emphasizes the importance of conservative treatment for a benign disease like diffuse recurrent hydatidosis. A muscle- or nerve-sparing attempt should always be performed and prolonged medical therapy is the mainstay of treatment. The risk/benefit ratio should always be analyzed in order to avoid extended surgical resections that may result in possible disability. Rare manifestations of neglected diseases are extremely important, especially for inexperienced physicians.
